# Machine learning dislocation density correlations and solute effects in Mg-based alloys

**DOI:** 10.1038/s41598-023-37633-9

**Published:** 2023-07-10

**Authors:** H. Salmenjoki, S. Papanikolaou, D. Shi, D. Tourret, C. M. Cepeda-Jiménez, M. T. Pérez-Prado, L. Laurson, M. J. Alava

**Affiliations:** 1 Department of Applied Physics, Aalto University, PO Box 11000, 00076 Aalto, Finland; 2 Present Address: NOMATEN Centre of Excellence, National Centre for Nuclear Research, A. Soltana 7, 05-400 Otwock-Swierk, Poland; 3 IMDEA Materials Institute, C/ Eric Kandel, 2, Getafe, 28906 Madrid, Spain; 4 Department of Physical Metallurgy, CENIM-CSIC, Avda. Gregorio del Amo 8, 28040 Madrid, Spain; 5 Computational Physics Laboratory, Tampere University, P.O. Box 692, 33014 Tampere, Finland

**Keywords:** Mathematics and computing, Physics, Condensed-matter physics, Statistical physics, thermodynamics and nonlinear dynamics, Techniques and instrumentation, Materials science, Structural materials, Techniques and instrumentation, Theory and computation

## Abstract

Magnesium alloys, among the lightest structural materials, represent excellent candidates for lightweight applications. However, industrial applications remain limited due to relatively low strength and ductility. Solid solution alloying has been shown to enhance Mg ductility and formability at relatively low concentrations. Zn solutes are significantly cost effective and common. However, the intrinsic mechanisms by which the addition of solutes leads to ductility improvement remain controversial. Here, by using a high throughput analysis of intragranular characteristics through data science approaches, we study the evolution of dislocation density in polycrystalline Mg and also, Mg–Zn alloys. We apply machine learning techniques in comparing electron back-scatter diffraction (EBSD) images of the samples before/after alloying and before/after deformation to extract the strain history of individual grains, and to predict the dislocation density level after alloying and after deformation. Our results are promising given that moderate predictions (coefficient of determination $R^2$ ranging from 0.25 to 0.32) are achieved already with a relatively small dataset ($\sim$ 5000 sub-millimeter grains).

## Introduction

Plastic deformation of crystalline materials is a problem of many length-scales. From the atomic level of dislocation core to the meso-scale collective dislocation dynamics, and ultimately to the grain boundary dynamics in polycrystals, dislocation mechanisms determine mechanical and physical properties. In single-crystalline hcp magnesium, the interplay between basal and non-basal slip mechanisms, leads to low strength and ductility, thus restricting possible applications. However, due to the compelling low weight of magnesium, improving the strength and ductility of Mg-based materials by alloying, is highly pursued^1^.

Meanwhile, materials informatics has become an emerging paradigm in the study and design of advanced materials^2–4^. Data science and machine learning tools can expedite, for instance, the experimental search for optimal compositions of Mg-based alloys with respect to target mechanical properties^5,6^. More generally, this quantitative perspective can give more insight into microstructural and local dislocation density evolution^7,8^. In polycrystals, machine learning can enable prediction of grain-wise properties from stress response^9–11^ to twin nucleation^12–14^ and, recently, graph-based representation of the granular structure has shown promise^11,15,16^.

**Figure 1:**
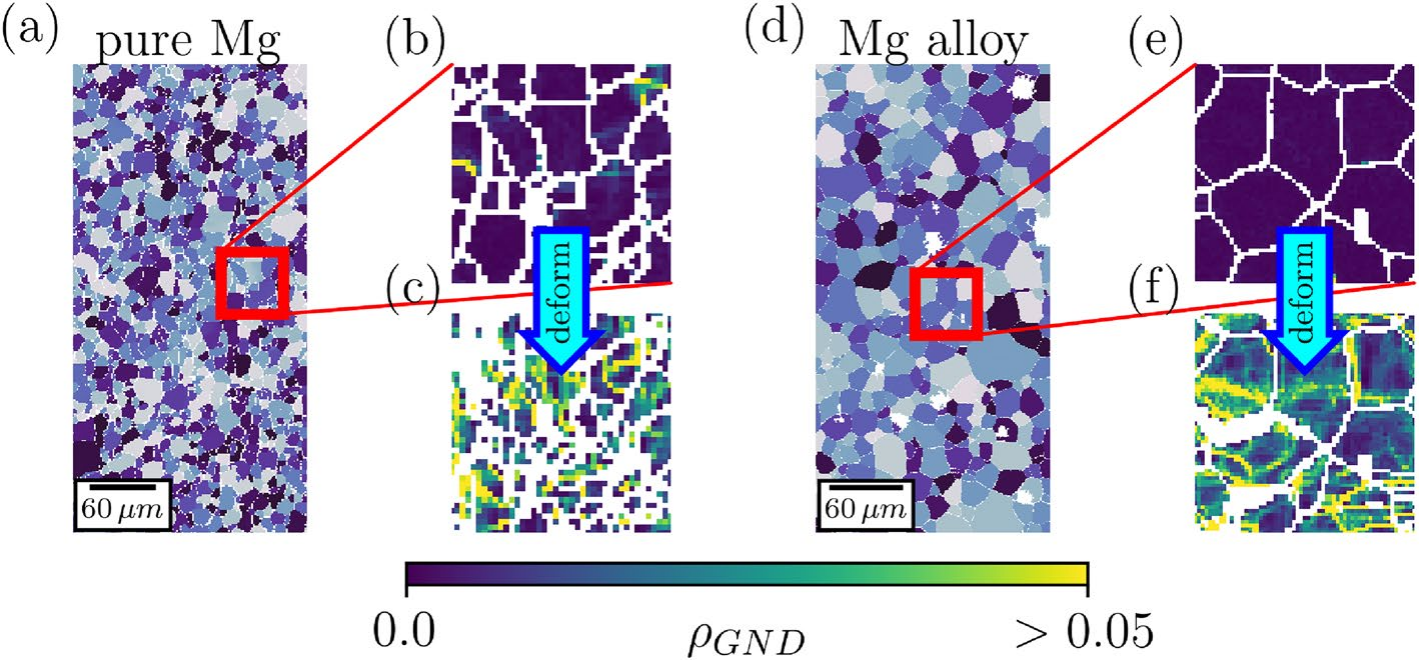
EBSD imaging for machine learning. (**a,d**) $225 \upmu \textrm{m} \times 450 \upmu \textrm{m}$ snapshots of the grain structure of the pure Mg and Mg alloy samples, respectively. The samples were characterised by their $\rho _{GND}$ before (**b,e**) and after deformation (**c,f**), see text for more information. The images in (**b,c,e,f**) are representative of the images used for machine learning purposes, in the four classes studied in this manuscript.

In the quest of improving the ductility and strength of Mg-based alloys, it is imperative to capture the precise mechanisms that dictate mechanical properties. In this context, this article promotes a data science approach towards the understanding of dislocation density evolution, the key component of mechanical response in advanced metals. We pursue this data science approach with respect to how common experimental protocols proceed. For this purpose, we compare EBSD images from pure Mg and a polycrystalline Mg–Zn alloy (2wt.% Zn) samples depicted in Fig. 1 which were originally introduced in^17^ (along with the sample preparation details). The dog-bone-shaped samples had final size with $\sim 3\,$mm thickness and $10\,$ mm gage length and the EBSD images covered an area of approximately $1.0 \times 0.7$  mm$^2$ with around 4000 and 6000 initial grains in the pure Mg and the alloy samples, respectively. The average grain size in both samples was similar, $\approx 13\, \upmu m$. The samples were also deformed to 10% strain and low-resolution EBSD images were produced post-deformation, thus yielding four classes, total, of EBSD images, the core of the investigations in this work.

We analyze dislocation densities together with grain boundary properties in the two samples before and after tensile tests with a true strain rate of $10^{-3}$  s$^{-1}$, reaching approximately $10\%$ strain. Notice that we look at the samples post-mortem at zero elastic strain. The aim for the study is twofold: First, to deduce the strain history of single grains from measured dislocation densities^18–20^. And second, to formulate predictions of dislocation density evolutions on the granular level^21,22^, due to the combined effects of deformation and alloying. Especially the latter is vital in deciphering possible dislocation density hotspots that may have major impact on work hardening. However, due to having access to only few low-resolution EBSD images, the scope of our study is to show the possibilities of the mentioned data science approaches while more detailed implementation is left for future work.

## Methods

The EBSD maps use a field emission gun (FEG) SEM (Helios NanoLab 600i, FEI) furnished with an HKL EBSD system, a CCD camera and the software package Channel 5.0 for data acquisition and analysis. The measuring conditions include parameters of 15 kV and 2.7 nA. The sample holder was tilted at 70$^\circ$ with respect to the horizontal in order to increase the backscattered signal.

The EBSD data was analyzed through the use of the MTEX toolbox for MATLAB^23^ and was further analyzed using cluster analysis and dimensionality reduction through sklearn Python libraries^24^. MTEX is able to infer the grain structure and local lattice orientation tensor $\kappa$ from the EBSD image, although the latter excludes the $\kappa _{i3}$ components which would require intrusive measurements. The MTEX-inferred $\kappa$ tensor across the material surface is the information that is used for this work ^23^. With the local orientation tensor, one is able to obtain the Nye’s dislocation tensor $\alpha$^25^,

1 $$\begin{aligned} \alpha _{ij} = \kappa _{ji} - \delta _{ji} \kappa _{kk}, \end{aligned}$$

where $\delta _{ji}$ is the Kronecker delta function. Furthermore, $\alpha$ is connected to the geometrically necessary dislocation (GND) density $\rho _{GND}$^26^

2 $$\begin{aligned} \rho _{GND} = \frac{1}{b} ||\alpha ||_1 \end{aligned}$$

where *b* is the magnitude of the Burgers vector. Notably due to the missing components of $\kappa$, the presented values here express only a lower limit of e.g. $\rho _{GND}$^27^. Moreover, the estimated $\rho _{GND}$ from the pure Mg and Mg alloy were not directly comparable, as the imaging step size differed between the two samples^28^.

**Figure 2:**
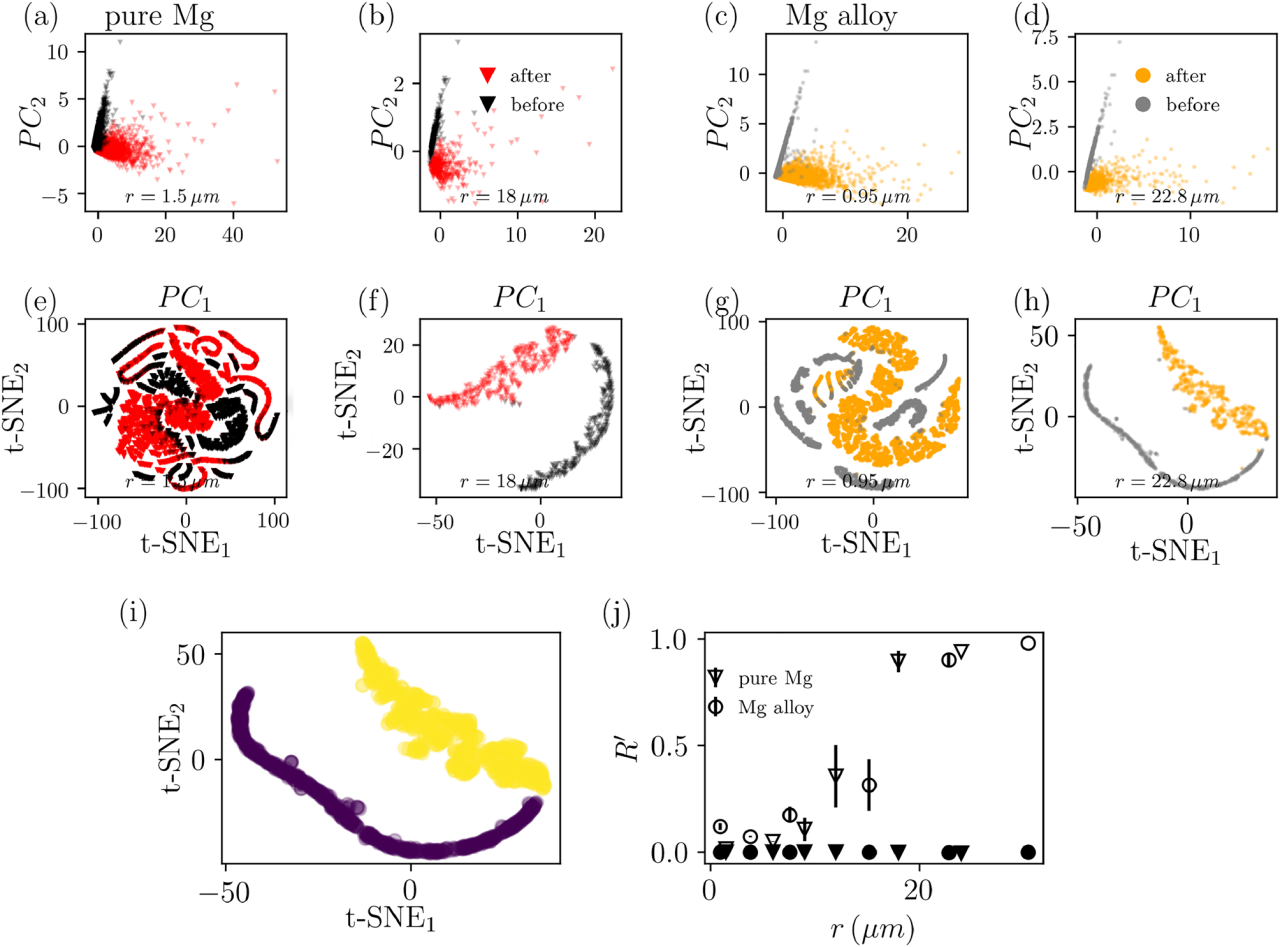
Clustering and unsupervised machine learning for EBSD images, using dislocation densities. (**a,b**) Pure Mg grains from both before and after deformation in the space of the two first principal components (PC) with $r=1.5 \,\upmu$m and $r=18 \, \upmu$m. (**c,d**) Mg alloy grains in PC space with $r=0.95 \, \upmu$m and $r=22.8 \, \upmu$m. (**e**–**h**) Same grains with t-SNE. (**i**) Example result of agglomerative clustering of grains seen in (**h**). (**j**) Adjusted Rand index $R'$ as a function of *r* after PCA (closed symbols) and t-SNE (open symbols). Due to t-SNE having stochastic nature, the results were obtained by repeating the dimensionality reduction and clustering three times per value of *r* and the errorbars show the standard deviation.

We characterized every grain by the sum of the GND density over the pixels of the grain. In addition, we computed parameters capturing local GND structures, i.e.

$$\begin{aligned} d\rho _{GND,1}(r)= & {} \sum \sqrt{(\rho _{GND}(x,y) - \rho _{GND}(x+r,y) )^{2} },\\ d\rho _{GND,2}(r)= & {} \sum \sqrt{(\rho _{GND}(x,y) - \rho _{GND}(x,y+r) )^{2} }, \\ d\rho _{GND,3}(r)= & {} \sum \sqrt{(\rho _{GND}(x,y) - \rho _{GND}(x+r,y+r) )^{2} }, \end{aligned}$$

where *r* is the shift in pixels times the step size. When varying the parameter *r*, grains with dimensions smaller than *r* are excluded from the analysis as the features are not defined for those grains. With grain size *s*, these parameters form a vector of five elements describing every grain: (*s*, $\rho _{GND}$, $d\rho _{GND,1}$,$d\rho _{GND,2}$ and $d\rho _{GND,3}$). In Fig. 2, the grain features for both pure and alloy Mg are illustrated with reduced dimensionality by principal component analysis (PCA) (Fig. 2a–d) and t-distributed stochastic neighborhood embedding (t-SNE)^29^ (Fig. 2e–h) and varying *r*.

The linear transformation of the PCA does not distinguish the grains by their strain history in neither pure nor Mg alloy, but t-SNE and sufficiently large *r* the grains form two distinct clusters with good correspondence with the EBSD image they were extracted from.

## Results

We quantified the distinguishability by first clustering the grains in the space of reduced dimensionality (e.g. Fig. 2i) and then measuring how well the found clusters coincide with the actual labels (before and after loading). For clustering we used hierarchical agglomerative clustering (bottom-up) with single linkage distance measure and the correspondence between the clusters and actual labels was measured with adjusted Rand index $R'$,^30,31^

3 $$\begin{aligned} R' = \frac{R - R_{random}}{1 - R_{random}}, \end{aligned}$$

where *R* (Rand index) is the fraction of correctly labeled pairs of datapoints (either correctly same or different labels) of all possible pairs and $R_{random}$ is the expected *R* with totally random clustering (i.e. $R'=1$ perfect correspondence; $R'=0$ random clustering). Fig. 2j shows $R'$ as a function of *r* for both samples and the results are similar: With $r\approx 18 \, \upmu$m the clustering achieves close to perfect success. This arises from two effects, namely the smaller grains that are harder to interpret get excluded from the analysis and long range dislocation structures emerge. Thus, as previous simulation results have shown, the strain history of grains is distinguishable in the Mg samples from the GND density evolution^18^. Both the dimensionality reduction and clustering were implemented with *scikit-learn*^24^.

**Figure 3:**
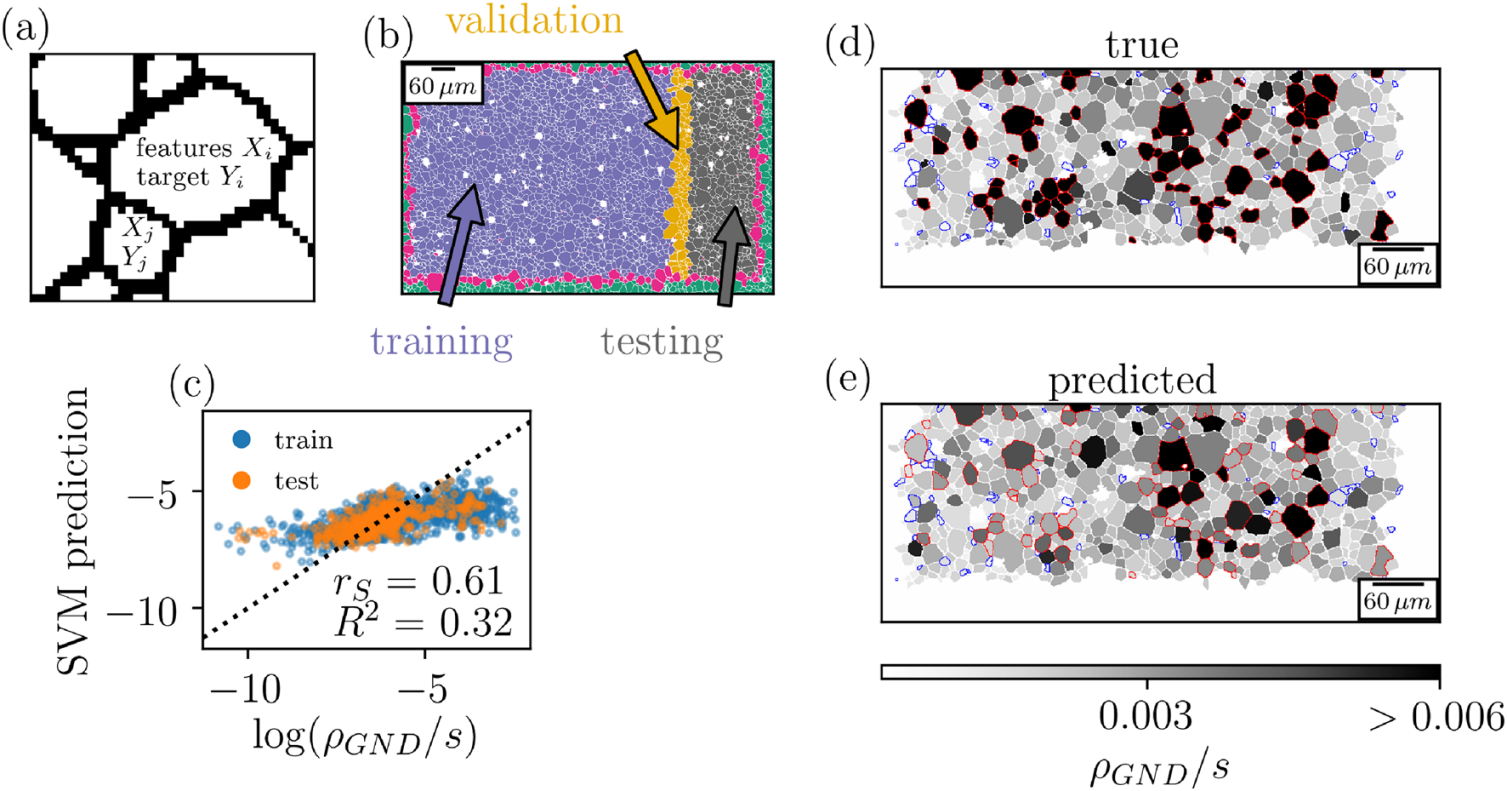
Predicting intra-grain dislocation contents in deformed specimens, using EBSD images. (**a**) Predicting grain-level dislocation density evolution starts by representing grains by their features $X_i$ before deformation and building the target by collecting $\rho _{GND}/s$ after deformation. (**b**) Train-test split of the grains in the alloy sample. The grains colored in red are bordering grains which were partially outside the image after loading and had target unavailable. (**c**) The SVM predicted versus the true values of $\log ( \rho _{GND}/s)$ for both the training and test grains. (**d,e**) Grains in the test set (axes transposed compared to (**b**)) colored by the true and predicted $\rho _{GND}/s$. The red (blue) highlighted grains correspond to those in top (bottom) 10% of all data.

In addition to distinguishing the strain history, the data set provided the classic setup for supervised prediction of properties of the sample—e.g. the evolution of $\rho _{GND}$—from the initial image before loading. To elucidate, Fig. 3a is a schematic showing grain *i* for which we can compute features $X_i$ before loading, similarly as above for the unsupervised clustering, and we can try to map the features to target value $Y_i$ which we set as $\log \rho _{GND}/s$ i.e. logarithm of the average GND density of the grain in the image after loading (more about data collection in Supplementary Note 1). Due to the noisiness in the EBSD image of the pure Mg sample after loading (Fig. 1c), we were unable to collect a proper target set for the sample and, thus, the supervised machine learning was done only with the alloy sample. We added simple features describing the grains and their neighbors to the feature set used above for clustering such as the orientation of the grain, average misorientation at the boundary and number of neighbors (full list of features for $\rho _{GND}$ prediction is found in Supplementary Table I). The sample was then divided into training, validation and testing grains as shown in Fig. 3b and, as we had only a single sample, we chose a considerably small validation set (5% of grains) to ensure as large training set (75%) as possible. Rest of the grains (20%) were used to test the model fit. The mapping was implemented with Support Vector Machine (SVM) (Supplementary Note 2), chosen due to the comparatively small number of hyperparameters; we have tried also other, more complex ML models such as artificial neural networks, obtaining similar results. We note that the number of grains in the dataset is relatively small, and hence the limiting factor might not be the ML model but rather the limited amount of training data.

Figure 3c illustrates the true versus predicted $\log \rho _{GND}$. The correlation between the target and the prediction are moderate as implied by the coefficient of determination $R^2 = 0.32$ and Spearman rank correlation coefficient $r_{S}=0.61$ for the test grains. Moreover, Fig. 3d–e shows the predicted $\rho _{GND}/s$ compared to the true values as a map of the test grains. Clearly the SVM is able to find hotspots with high $\rho _{GND}$ with good accuracy although some errors exist and for some cases the high densities are predicted for neighboring grains (e.g. bottom left edge grain with high true $\rho _{GND}/s$). Overall, the results are remarkable considering only a single sample was used.

Obviously our prediction success suffers from some properties of the dataset and used features. Firstly, our tracking algorithm does not always find the exact pixels of the image after loading corresponding to certain grain in the initial image. Also the noisiness and missing $\rho _{GND}$ pixels in the after image cause imprecision to the target values (Supplementary Note 3). And more importantly, the defined grain features do not capture all relevant characteristics of the sample: they neglect most information about the grain boundaries between the neighboring grains and the well-known grain boundary effect of impeding dislocation motion^32,33^.

**Figure 4:**
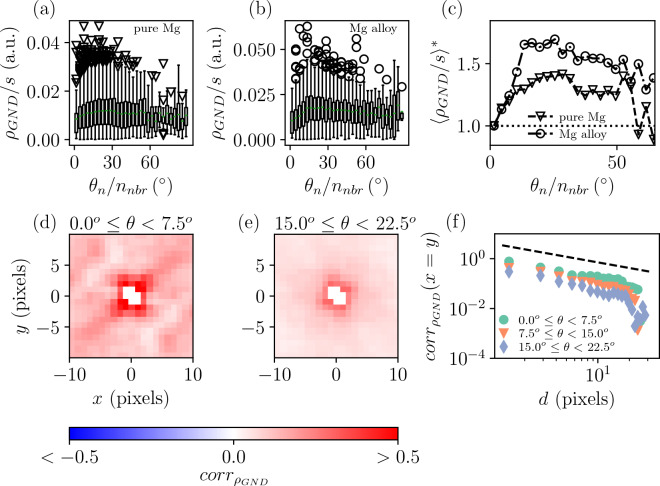
Dislocation correlations, extracted from EBSD images. (**a,b**) Boxplots showing the distribution of grain-wise $\rho _{GND}/s$ after deformation binned according to average misorientation at boundary $\theta _n / n_{nbr}$, i.e. the total misorientation summed over neighbor grains divided by the number of neighbors, for pure and Mg alloy, respectively. The green lines show the distribution average. (**c**) The average seen in the boxplots as a function of $\theta _n / n_{nbr}$ scaled to start from unity. (**d,e**) Correlation (Eq. 4) of pixel $\rho _{GND}$ in Mg alloy sample after deformation across grain boundaries (*x*, *y* the distance from the boundary) for neighboring grains with misorientation $0^\circ \le \theta <7.5^\circ$ or $15^\circ \le \theta <22.5^\circ$, respectively. (**f**) The correlation along the diagonal $x=y$ for three consecutive misorientation ranges. The dashed line shows $d^{-3/4}$ as a guide to eye.

For instance $\rho _{GND}/s$ shows visible dependence on the average misorientation $\langle \theta \rangle$ at the grain boundary for grains after loading in both samples as seen in Fig. 4a–c. The data shows that the relative increase in $\rho _{GND}/s$ vs $\langle \theta \rangle$ seems to be steeper in the alloyed sample, although the above mentioned difference in the imaging protocol (step size) can have an effect too. Moreover, Fig. 4d–f presents analysis of $\rho _{GND}$ correlation across grain boundaries,

4 $$\begin{aligned} corr_{\rho _{GND}}(x,y) = \frac{ \langle (\rho _{GND}(x', y') - {\overline{\rho }}_{GND}) (\rho _{GND}(x'+x, y'+y) - {\overline{\rho }}_{GND}) \rangle }{ \langle \rho _{GND} - {\overline{\rho }}_{GND} \rangle ^2 }, \end{aligned}$$

where the coordinate $(x', y')$ lies inside grain *i* and $(x'+x, y'+y)$ lies inside grain *j* which is a neighbor of *i*, in grains of the Mg alloy after loading. The data is computed for grain pairs (where grains with size smaller than 64 pixels were omitted) with misorientation angle in a given range. As is evident from comparing the low-angle grain pairs with $0^\circ \le \theta <7.5^\circ$ in (d) and high-angle grain pairs with $15^\circ \le \theta <22.5^\circ$ in ( e), the correlations diminish when the misorientation angle increases. More generally, Fig. 4f illustrates the decay of the correlation (along $x=y$) for three subsequent ranges of $\theta$. The overall decay somewhat follows $corr_{\rho _{GND}}(x=y) \propto d^{-3 / 4}$ where *d* is the distance from the neighbor grain. And the difference in the misorientation angle between the grains shifts the correlation curve, i.e. correlations decrease when $\theta$ increases and vice versa

**Figure 5:**
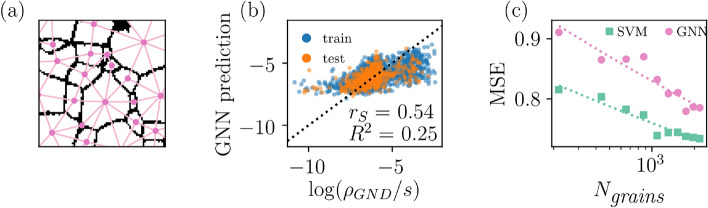
Prediction of post-deformation dislocation densities using grain-based graph neural networks. (**a**) Schematic showing the grain structure represented as a graph. Every graph forms a node and grains with common boundary have a connection edge. The node features correspond to the $X_i$ used for SVM while the edge features $E_{ij}$ include e.g. misorientation between the grains. (**b**) Graph network prediction versus the true value of $\log (\rho _{GND}/s)$ for training and test sets. (**c**) The mean squared error between the true and predicted values of test set as a function of number of grains $N_{grains}$ used to train both SVM and GN models.

To incorporate the grain boundary effect into the prediction problem, we formed a graph of the granular structure, as in Fig. 5a, collected features $E_{ij}$ characterising the boundaries between neighbouring grains (e.g. misorientation; see Supplementary Table II) and applied graph networks (GN) to predict $\log \rho _{GND}/s$^34^. The GN architecture followed the encode-process-decode network which has been used on e.g. glassy systems with some success^34,35^ (More details on the GN training procedure and results are presented in Supplementary Note 4). Figure 5b shows the true versus GN predicted $\rho _{GND}$. Conversely, the goodness of the model prediction is slightly worse than with SVM and without grain boundary properties, $R^2=0.25$ and $r_{S}=0.54$. But, as mentioned earlier, the data set has its limitations: Fig. 5c presents the learning curves for SVM and GN obtained by fitting the models with reduced training set (i.e. excluding part of the training grains) and measuring the mean squared error (MSE) for the test set. The figure highlights the smallness of the training set as the prediction success has not converged. By fitting a decreasing power law, $MSE \propto N_{grains}^{-\alpha }$, to the learning curves, it seems that the GN loss is decreasing with steeper slope and, therefore could outperform SVM with proper data set encompassing multiple samples.

## Discussion

In conclusion, we have applied machine learning methods to study and predict dislocation density evolution in Mg samples. Observing only the GND density obtained by low-resolution EBSD, we were able to distinguish the strain histories of individual grains. Moreover by using the grain-wise information of the Mg alloy sample before tensile loading, we trained SVM and GN to predict GND density after loading. Both SVM and GN produced predictions with adequate success and, although the SVM outperformed the GN which used also the grain boundary information, the GN showed possibility of more significant improvement with larger datasets which gives a natural direction for future research. In addition, for future studies of GNDs (as well as statistically stored dislocations), it might be interesting to explore the use of methods which probe a larger depth of the material, such as microLaue diffraction^36^. Overall, the used machine learning methods show promise in the study of the plastic deformation on the granular level, and in the long run, machine learning can assist in optimising granular properties to achieve desired material properties. Here, we have shown this by looking at two cases and our results show how these samples exhibit different signatures. On one hand, the strain history is distinguishable regardless of the alloying procedure. But on the other hand, some differences are seen in the grain boundary effect between the pure Mg and alloy cases as the dislocation density inside a grain increases more rapidly with the average misorientation at the boundary in the alloyed sample.

## Supplementary Information

Supplementary Information.

## Data Availability

The data supporting the findings of this paper are available from the corresponding author upon reasonable request.
