# Supplementary material for: Machine learning dislocation density correlations and solute effects in Mg-based alloys
Source: Sci Rep. 2023 Jul 10;13:11114. doi: 10.1038/s41598-023-37633-9 (PMC10333208; doi:10.1038/s41598-023-37633-9)
Supplement: Supplementary file 1 — Supplementary Information. [file 41598_2023_37633_MOESM1_ESM.pdf]

Supplementary information to "Machine learning dislocation density correlations and solute effects in Mg-based alloys"

Salmenjoki et al.

Supplementary Note 1: Data acquisition To collect the corresponding grain data before and after loading, we started by finding the correct coordinates of the same position in the both images. We started by computing a map of mean squared difference (MSD) between the pixel orientation of 50×50 pixel sub images between before and after loading data. Then we collected the MSD map into a coordinate map by first retrieving the coordinates of the minimum in the MSD map for every sub image and then (median) filtering the coordinate map to reduce noise and achieve a smooth coordinate map between the two images. Obviously, the correspondence of the grains are not perfect as the after image has been deformed versus the before image but this way we get adequate matches as illustrated by the examples in Supplementary Fig. 1. But this way, we were able to collect a dataset where we have the features of the initial grain and the ρ_{GND} in the pixels of the post-deformation image corresponding to the initial grain.

However as the figure shows, we lose some pixels that go missing due to new forming grain boundaries, inaccuracy in finding the correct pixels or changing shape of the grains. To counter the first, i.e. new grain boundaries, we took twinning into account by merging grains with grain boundaries matching the twinning misorientation in the after image (following the procedure documented in the MTEX manual [1]). This way we were able to reduce the number of missing pixels in some cases but, as noted in the original publication presenting the data, twinning is not dominant deformation mechanism in the studied samples [2]. One example of a twinning grain is illustrated in Supplementary Fig. 2.

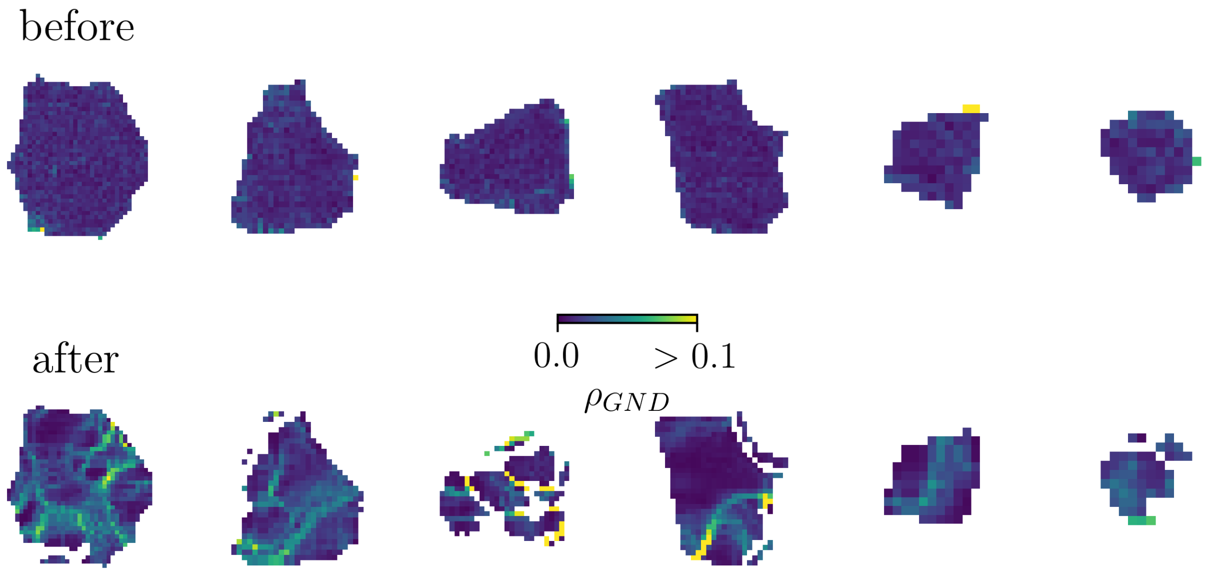

Supplementary Figure 1. Examples of grains from the alloy sample before (top row) and after (bottom row).

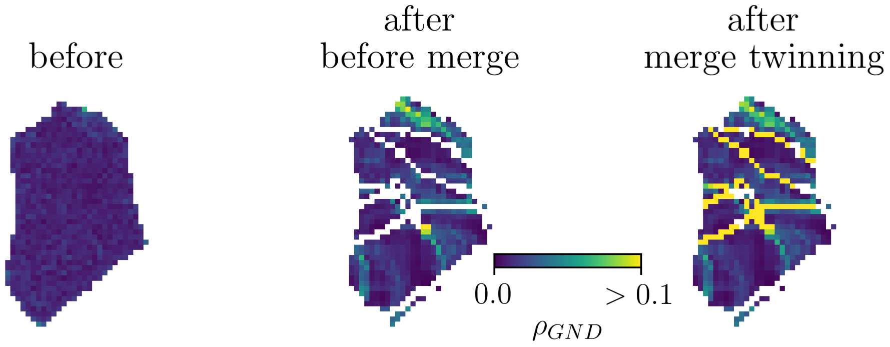

Supplementary Figure 2. An example grain in the alloy sample before loading and after loading without and with merging the twinning grains.

Supplementary Note 2: grain features used The processing of the imaging data is done by fixing a number of geometric parameters in the following table.

TABLE I. **Extracted grain features X from the image before loading.**

size s	circumference
max width	max height
ρ_{GND}/s	$d\rho_{GND,1}(4\,\mu m)$
$d\rho_{GND,2}(4\,\mu m)$	$d\rho_{GND,3}(4\,\mu m)$
orientation parameters ϕ_1, Φ, ϕ_2	Grain Average Misorientation (GAM)
sum of misorientations between nbrs θ_n	number of neighbors n_{nbr}
average misorientation θ_n/n_{nbr}	Grain Orientation Spread (GOS)

Supplementary Note 3: SVM training details The SVM was implemented with the *scikit-learn* library [3]. Our SVM used the radial basis function kernel. The ϵ hyperparameter, "tube" was set to 0.25 and the penalty parameter $C \approx 1.5$ was deduced by finding the optimal value according to the loss on validation grains as illustrated in Supplementary Fig. 3.

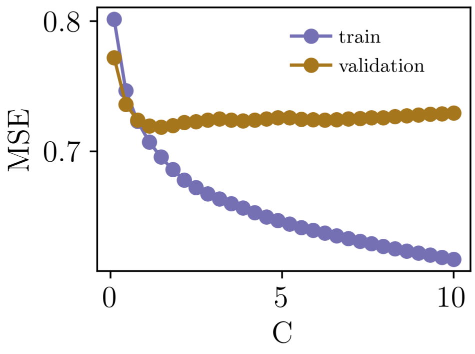

Supplementary Figure 3. MSE error of SVM prediction for training and validation sets as a function of hyperparameter C.

Supplementary Note 4: Missing pixels in the EBSD image and impact on the SVM predictions As discussed in the main text and above in Supplementary note 1, finding one-to-one correspondence of grains in images before and after loading has several challenges. One is the increasing amount of noisy pixels in the after image. This arises at least partially from new, forming grain boundaries [4], but also drastic local orientation differences lead to

missing values of ρ_{GND} in the after image.

Due to the missing pixels, the target value for which the SVM mapping is done contains noise and it affects the SVM prediction success. To elaborate, Supplementary Fig. 4 shows the absolute error versus the fraction of missing pixels in the image after loading (i.e. 0 all pixels found, 1 all pixels missing) for single grain predictions along with the average curve. And as expected, the error of SVM output increases with the number of missing pixels and does so drastically when approaching 1.

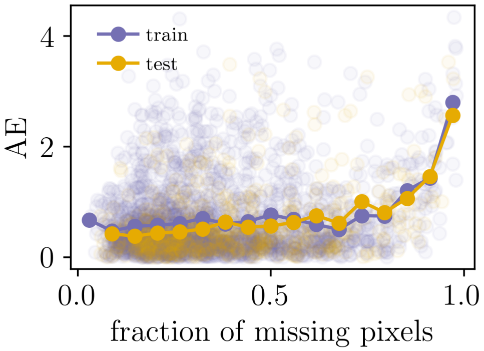

Supplementary Figure 4. Absolute error between the true and predicted values versus the fraction of missing pixels in the grain after loading for single grains (points) and the mean (line).

Supplementary Note 5: grain boundary fetures used

TABLE II. **Extracted grain boundary features E from the image before loading.**

misorientation θ
length of grain boundary
direction vector $\mathbf{g} = (g_1, g_2)$ pointing from one grain center to another

Supplementary Note 6: GN training details For the GN architecture, we used the encode-process-decode machine [5]. The idea of the model is to use the features of nodes (grains) and edges (grain boundaries between neighboring nodes) to predict node-wise variable (the target is $\log \rho_{GND}/s$ after loading). First, the model encodes the data, both node and edge features, into latent space and then processes the data by passing the latent representation of the features to neighbor nodes. The number of processing steps then decides on how 'far' the interactions are considered to apply (e.g. with three processing steps a node receives the data of all nodes reachable in three steps via the message-passing). Finally after the processing steps, the processed data is decoded from the latent space back to the desired form (i.e. the target).

We implemented the GN with the Deepmind's Graph Nets library [5]. For training, we applied the same idea as e.g. in [6] where the number of processing steps is fixed to some value but the GN is encouraged to solve the problem with as few steps as possible. We used five steps which is quite many considering that not necessarily grains that many jumps apart affect each other. And indeed, Supplementary Fig. 5a shows the training and validation loss during the training and the smallest validation loss is achieved with three processing steps. Other parameter values used for our model: 32 latent parameters for nodes, 16 latent parameters for edges, two hidden layers in encoder, decoder and processor. Learning rate was set to $5 \cdot 10^{-6}$ and the training was conducted with the early-stopping criterion, i.e. according to Supplementary Fig. 5a, the optimal GN was obtained after approximately 5000 epochs where the validation loss was at its minimum.

To further elaborate what the GN has learned at the end of the training, Supplementary Figs. 5b-c show the latent representation of the nodes after three processing steps in the space of reduced dimensions (t-SNE). In Supplementary Fig. 5b, the coloring is according the target value the GN is trying to learn and there are clearly separated regions with high ρ_{GND}/s and low ρ_{GND}/s grains. Furthermore in Supplementary Fig. 5c, the coloring is according to twinning / not twinning and again some separation can be seen in the latent space (i.e. grains that are more inclined to twinning).

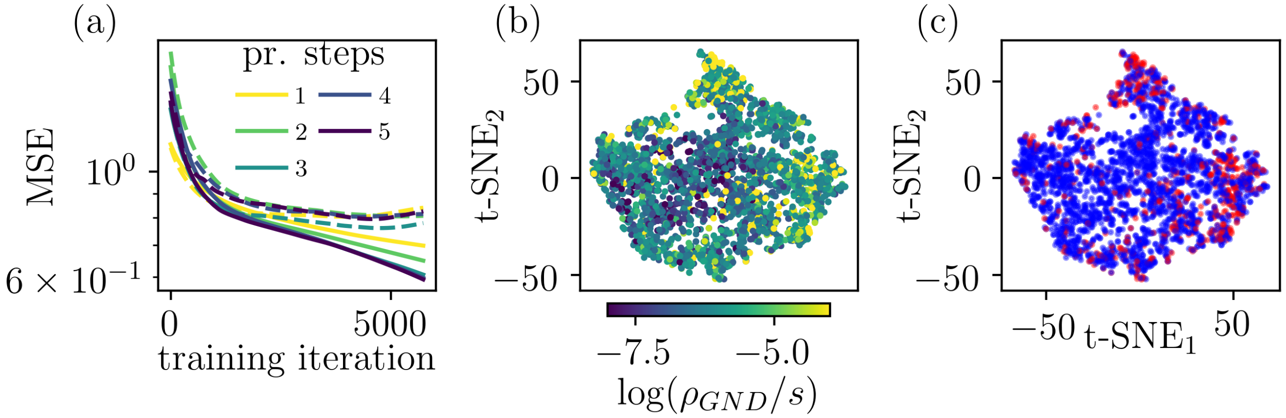

Supplementary Figure 5. (a) Training (solid) and validation (dashed lines) loss during the GN training with different number of processing steps. (b) Latent representation of the nodes after three processing steps colored by the target value $\log \rho_{GND}/s$. (c) Latent representation of the nodes now colored by label twinning (red) / not twinning (blue).

---

[1] F. Bachmann, R. Hielscher, H. Schaeben, in: Solid State Phenomena, volume 160, Trans Tech Publ, pp. 63–68.

[2] D. Shi, M. Pérez-Prado, C. Cepeda-Jiménez, Acta Materialia 180 (2019) 218–230.

[3] F. Pedregosa, G. Varoquaux, A. Gramfort, V. Michel, B. Thirion, O. Grisel, M. Blondel, P. Prettenhofer, R. Weiss, V. Dubourg, J. Vanderplas, A. Passos, D. Cournapeau, M. Brucher, M. Perrot, E. Duchesnay, Journal of Machine Learning Research 12 (2011) 2825–2830.

[4] P. Lehto, Ultramicroscopy 222 (2021) 113203.

[5] P. W. Battaglia, J. B. Hamrick, V. Bapst, A. Sanchez-Gonzalez, V. Zambaldi, M. Malinowski, A. Tacchetti, D. Raposo, A. Santoro, R. Faulkner, et al., arXiv preprint arXiv:1806.01261 (2018).

[6] V. Bapst, T. Keck, A. Grabska-Barwińska, C. Donner, E. D. Cubuk, S. S. Schoenholz, A. Obika, A. W. Nelson, T. Back, D. Hassabis, et al., Nature Physics 16 (2020) 448–454.
